# Liver regeneration after extensive hepatectomy in rats: effect of preoperative chemotherapy with intravenous 5-fluorouracil

**DOI:** 10.1590/acb370901

**Published:** 2022-11-28

**Authors:** Marciano Anghinoni, Edimar Leandro Toderke, Thaísa Sami Nakadomari, Tiago Kuchnir Martins de Oliveira, Felipe Pedrotti Locatelli, Jorge Eduardo Fouto Matias

**Affiliations:** 1 Fellow MSc. Universidade Federal do Paraná – Departamento de Cirurgia – Pós-Graduação em Clínica Cirúrgica – Curitiba (PR), Brazil.; 2 Fellow PhD. Universidade Federal do Paraná – Departamento de Cirurgia – Pós-Graduação em Clínica Cirúrgica – Curitiba (PR), Brazil.; 3 General Surgeon. Hospital São Vicente – Curitiba (PR), Brazil.; 4 General Surgeon. Hospital Sugisawa – Curitiba (PR), Brazil.; 5 PhD, Associate Professor. Universidade Federal do Paraná – Departamento de Cirurgia – Pós-Graduação em Clínica Cirúrgica – Curitiba (PR), Brazil.

**Keywords:** Liver Regeneration, 5-Fluorouracil, Hepatectomy, Rats

## Abstract

**Purpose:**

To evaluate the effect of preoperative intravenous chemotherapy with 5-fluorouracil on liver regeneration in an experimental model of major hepatectomy in rats.

**Methods:**

Wistar rats were divided into two groups of 20 animals each and submitted to 70% hepatectomy 24 h after intravenous injection of 5-fluorouracil 20 mg/kg (fluorouracil group, FG) or 0.9% saline (control group, CG). After hepatectomy, each group was subdivided into two subgroups of 10 animals each according to the day of sacrifice (24 h or 7 days). Liver weight during regeneration, liver regeneration rate using Kwon formula, and the immunohistochemical markers proliferating cell nuclear antigen (PCNA) and Ki-67 were used to assess liver regeneration.

**Results:**

At early phase (24 h after hepatectomy) it was demonstrated the negative effect of 5-fluorouracil on liver regeneration when assessed by Kwon formula (p < 0.0001), PCNA analysis (p = 0.02). With regeneration process complete (7 days), it was possible to demonstrate the sustained impairment of chemotherapy with 5-fluorouracil on hepatocytes regeneration phenomenon when measured by Kwon formula (p = 0.009), PCNA analysis (p = 0.0001) and Ki-67 analysis (0.001).

**Conclusions:**

Preoperative chemotherapy with intravenous 5-fluorouracil negatively affected the mechanisms of liver regeneration after major hepatectomy in rats.

## Introduction

The treatment of secondary liver malignancies, particularly from colorectal cancer (CRC) has achieved improvements in recent decades due to both technical developments related to liver resections, such as associating liver partition and portal vein ligation for staged hepatectomy (ALPPS), and the principles of the multimodal approach, with new therapies such as radiofrequency ablation, chemoembolisation, and neoadjuvant chemotherapy (NAC), also known as preoperative chemotherapy. Such strategies, regardless their nature, are all based on the ability of the liver parenchyma to preserve regenerative capacity despite being infiltrated by neoplastic tissue and attacked by chemotherapy agents. Whenever possible, surgical resection of metastatic liver lesions from CRC should be recommended, as it is currently the most reliable therapy despite a non-negligible rate of recurrence. In CRC, preoperative chemotherapy has emerged as a cutting-edge treatment associated with improved survival rates by increasing the number of patients who can be submitted to successful surgical treatment^1^^,^ 2.

Progress in multimodal strategies, including NAC and the use of new drug schemes have opened new perspectives in the treatment of resectable, potentially resectable, and unresectable liver metastases^3^^,^ 4. Recently, the combination of these schemes with targeted molecular therapies has further increased response rates^5^^,^ 6.

Additionally, controversies persist regarding the real effects of NAC on resection results. Although recent studies have reported that NAC does not increase the risks of liver resection and post-surgical complications^7^^–^ 9, it is not well established when the risk of postoperative liver failure is greater in the different schemes of chemotherapy.

In addition, the effect of these drugs on the regeneration potential of the remaining liver parenchyma after hepatectomy is still unknown and may be influenced by multiple factors not adequately controlled in available clinical trials. The number of patients treated with neoadjuvant drug regimens to optimise resection has progressively increased. More aggressive treatments and new chemotherapy regimens with new drugs, alone or in combination, have increased the number of patients able to undergo resection of metastatic liver lesions from CRC.

Therefore, it is crucial to determine the effects of NAC on regenerating liver tissue. In order to assess the potential negative effects of preoperative chemotherapy on liver regeneration, this study evaluated the impact of preoperative intravenous 5-fluorouracil chemotherapy on an experimental model of extensive hepatectomy in rats.

## Methods

This study complied with the ethical standards established by the Brazilian College of Animal Experimentation and was approved by the Research Ethics Committee of Universidade Federal do Paraná, Health Sciences Sector, under the certificate CEP/SD: N. AN.021.004.09.12.

Male Wistar rats (*Rattus norvegicus albinus*) weighing 280–350 g were obtained from Institute of Technology of Paraná (Tecpar), Araucária, Brazil. The animals were kept four per plastic cage, with environment temperature control (22 ± 1 °C) and automated 12-h light-dark cycle. They received water and industrial rat chow (Nuvilab CR1) *ad libitum* throughout the experiment.

### Drugs and solutions

The solution of 5-fluorouracil at a concentration of 50 mg/mL was the drug used for chemotherapy in the study groups. For each administration, a dilution of 1 mL of the solution was prepared in 4 mL of 0.9% saline to obtain a final concentration of 10 mg/mL. Control groups received equal final volume of 0.9% saline only.

### Study groups

Forty animals were randomly divided into two groups:

The fluorouracil group (FG) comprised 20 animals administered a single intravenous injection of 5-fluorouracil, at a dose of 20 mg/kg weight, 24 h before 70% hepatectomy. After liver resection, the group was divided into two equal subgroups of 10 animals according to the day of sacrifice: 24 h after hepatectomy (FG_24h_) or 7 days after hepatectomy (FG_7d_).

The control group (CG) comprised 20 animals administered a single intravenous injection of 0.9% saline in equivalent volumes to the FG, 24 h before 70% hepatectomy. After liver resection, the group was divided into two equal subgroups of 10 animals according to the day of sacrifice: 24 h after hepatectomy (CG_24h_) or 7 days after hepatectomy (CG_7d_).

### Inhalation anaesthesia

For anaesthetic induction, the animals were placed in a closed plastic reservoir connected to the anaesthetic vaporiser device which delivered isoflurane at final concentration of 3.0%. After observing the loss of consciousness and muscle relaxation, they were kept under continuous inhalation anaesthesia with isoflurane through a plastic mask adapted to their heads and connected to the anaesthetic vaporiser delivering a 1.5% isoflurane final concentration and maintaining an oxygen flow of 2 L/min.

### Preoperative chemotherapy administration

After fasting for 12 h, each animal was subjected to anaesthetic induction, weighed on a precision scale, and identified on the tail. Under continuous inhalation anaesthesia, each animal underwent trichotomy at the base of the tail, which was fixed with a tourniquet, and the largest caudal vein was identified and punctured with a 24-G Abocath catheter. The needle was removed and a 1-mL syringe with the appropriate volume of solution to be administered was connected to the Abocath. The tourniquet was then released and the solution was injected slowly, making sure that the administration was taking place inside the vessel lumen.

### Extensive hepatectomy

All animals of all groups and subgroups were submitted to extensive hepatectomy 24 h after the administration of the intravenous solutions (5-fluorouracil or 0.9% saline). The technique performed was the 70% hepatectomy model described by Higgins and Anderson^10^, which includes resection of the median and left lateral hepatic lobes.

**Figure 1:**
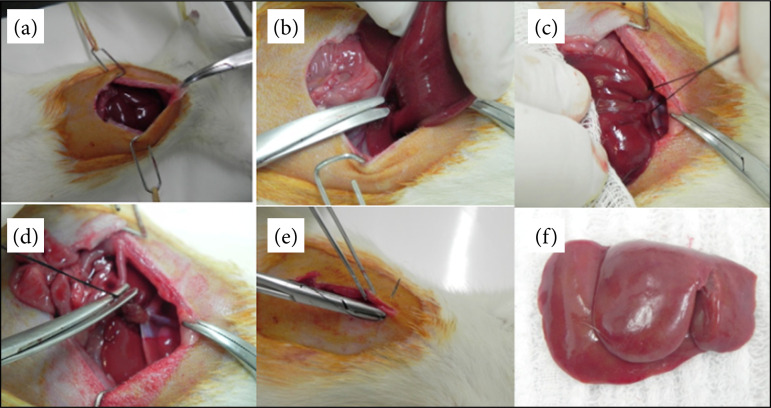
Technique of 70% hepatectomy. **(a)** Median laparotomy and installationof metallic retractors; **(b)** Liver dissection and ligamentous section; **(c)** and**(d)** Wire positioning, ligation, parenchyma section, and specimen removal;**(e)** Closure of the abdominal wall; **(f)** Resected liver lobescorresponding 70% of the liver parenchyma.

After anaesthetic induction, the animals were weighed on a precision scale, underwent abdominal wall trichotomy, and were fixed to the operating table. Under continuous inhalation anaesthesia, and after abdominal wall antisepsis with 10% PVPI, a 3-cm extension median laparotomy was performed. The peritoneal cavity was exposed using lateral metallic retractors and pulling the xiphoid process. After the section of falciform and left triangular ligaments, hepatectomy was performed using a single hepatic pedicular ligation with 2.0 cotton, with the left lobe and median lateral lobe being resected. The collected liver lobes were weighed and discarded. The abdominal wall was then sutured with 3.0 nylon thread and the animals returned to their cages (Fig. 1).

Depending on the study subgroup, sacrifice and collection of the remaining liver were performed on the first or seventh day after partial hepatectomy. The animals were sacrificed by lethal dose of inhaled ether, weighed, and fixed on the operating table. A relaparotomy was performed and the remaining liver was completely resected and weighed for posterior assessment of liver regeneration rate. A portion of the superior right lobe was sampled, fixed in 10% buffered formalin, and sent for immunohistochemical studies (PCNA and Ki67).

### Assessment of liver regeneration

#### Liver regeneration rate

The mathematical formula proposed by Kwon in 1990 was used to assess regeneration by liver mass criterion^11^. The liver regeneration rate after a 70% hepatectomy was calculated from Eq. 1:


$$\text{Hepatic regeneration rate}(\%)=\frac{D}{E}\times 100$$ (1)

where (*D*) represents liver weight per 100 g of body weight at the sacrifice day and *E* (*R*/0.7) represents estimated liver weight per 100 g of body weight at the day of 70% liver resection, which was calculated from the weight of the left lobe and the median lateral lobes resected (*R*). The body weight of all animals was evaluated in the first day of pretherapy, before the hepatectomy and in the sacrifice day in all subgroups.

#### Immunohistochemical markers: PCNA and Ki-67

The cell proliferation markers proliferating cell nuclear antigen (PCNA) and Ki-67 were used for immunohistochemical evaluation of liver regeneration. PCNA, a nuclear protein and factor of the DNA polymerase delta enzyme, is essential for DNA synthesis and cell replication. The Ki-67 antigen (or MKi67), a protein associated to the transcription of ribosomal RNA, is present in the nucleus of cells during mitosis and all phases of the cell cycle.

Primary anti-PCNA and anti-Ki-67 were used. Formalin-fixed, paraffin-embedded tissues were cut into 4-mm sections and mounted on slides. The avidin-biotin-peroxidase complex immunohistochemical method was performed in the samples. Slides were heated at 60° C for 60 min, deparaffinized in xylene, and then rehydrated in alcohol. Endogenous peroxidase was blocked by incubation in 0.3% hydrogen peroxidase in methanol, and slides were rehydrated and washed in phosphate buffered saline (PBS), pH 7.4 for 15 min. Sections were then blocked with 10% normal goat serum in PBS with 1% bovine serum albumin for 15 min. The blocking serum was decanted, and the primary antibodies anti-PCNA (Monoclonal, code M0879, Dako, Carpinteria, USA) e anti-Ki-67 (Monoclonal, code NCL-Ki-67-MM1, Clone MM1, Novocastra, USA) were applied, respectively, in PBS with 1% bovine serum albumin for 16h at 4 °C. Slides were washed in PBS with 1% Tween 20 for 10 min. After three PBS rinses, biotinylated goat anti-rabbit immunoglobulin (Vector Labs, Burlingame, CA) at a dilution of 1/500 was applied for 30 min at room temperature. After, another PBS rinse, avidin DH:biotinylated horseradish peroxidase complex (Vector Labs) was applied for 30 min. After a final PBS rinse, the tissue sections were reacted with 0.06% diaminobenzidine (Sigma) for 5 min, rinsed, counter-stained with haematoxylin, dehydrated with graded alcohols, cleared xylene, and coverslipped with Permount. The sections were analysed at a magnification of 400× and photomicrographs were performed using a Nikon Microphot system (Tokyo, Japan). Immunohistochemical staining positivity was identified in areas with brownish pigmentation. Positive and negative controls were used. Individual random analysis of the slides was performed while blinded to the group assignment. The number of stained nuclei in 100 hepatocytes at a random slide area was counted for each animal in each subgroup. Hepatocyte nuclei with moderate or strong brown colour were considered positive.

### Statistical analysis

Statistica version 6.0 was used to perform statistical analyses. The normal distribution (Gaussian) prerequisite was determined to choose the statistical tests. For nonparametric data, comparison analysis was performed using Mann–Whitney test. The parametric data analyses used Student t test. In all statistical tests, the significance level was p ≤ 0.05.

## Results

### Body weight

As a general trend, the animals lost weight throughout the experiment. However, no significant difference in body weight was found between control and fluorouracil subgroups throughout the experiment, demonstrating no interference of chemotherapy on lost body weight, even in the longer subgroups of 7 days (Table 1).

**Table 1:** Body weight comparisons between control (CG) and 5-fluorouracil (FG) subgroups at chemotherapy day (initial), at the hepatectomy day and sacrifice.

Subgroup	Initial (g)			Hepatectomy (g)			Sacrifice (g)	
	Average (SD)	p		Average (SD)	p		Average (SD)	p
CG_24h_	330.2 ± 13.6	0.17		314.6 ± 9.6	0.68		290.6 ± 11.4	0.57
FG_24h_	320.6 ± 16.6			316.7 ± 15.3			294.4 ± 17.2	
CG_7d_	324.6 ± 11.4	0.46		328.7 ± 13.3	0.72		328.2 ± 18.5	0.88
FG_7d_	329.0 ± 14.7			331.1 ± 13.3			327.1 ± 15.0	

SD: standard deviation; p: level of statistical significance by Student’s t test.

### Liver weight

Concerning the changes of liver mass throughout the study, it was possible to demonstrate evident influence of chemotherapy on liver weighing during the experiment. First, at the day of hepatectomy (24 h after chemotherapy), estimated liver mass of fluorouracil subgroups were higher than the control subgroups, significantly superior between 24 h subgroups’ comparison (p = 0.03). Second, at the sacrifice day (24 h or 7^th^ day), total regenerated liver weigh of fluorouracil subgroups was significantly lower than the control subgroups, completely reversing the initial situation, even in the earliest subgroups (24 h), suggesting negative impact of chemotherapy on liver regeneration. Finally, identical significant results were obtained when the same comparisons were evaluated using the regenerated liver weight to body weight ratio (Table 2).

**Table 2:** Intragroup (24 h vs. 7 days) and intergroup (control vs. fluorouracil) comparisons of liver weight **(g)** at hepatectomy and sacrifice days and regenerated liver to the body weight ratio.

Subgroup		Estimated liver weight (g) at hepatectomy			Regenerated liver weight (g) at sacrifice			Regenerated liver weight (g) to body weight ratio	
		Average (SD)	p		Average (SD)	p		Average (SD)	p
FG_24h_		9.04 ± 0.77	0.05		7.29 ± 0.62	0.0001		0.0248 ± 0.0017	0.0001
FG_7d_		9.64 ± 0.51			12.62 ± 0.98			0.0386 ± 0.0032	
CG_24h_		8.32 ± 0.64	0.01		8.65 ± 0.65	0.0001		0.0298 ± 0.0017	0.0001
CG_7d_		9.26 ± 0.86			13.92 ± 0.91			0.0425 ± 0.0031	
FG_24h_		9.04 ± 0.77	0.03		7.29 ± 0.62	0.0001		0.0248 ± 0.0017	0.0001
CG_24h_		8.32 ± 0.64			8.65 ± 0.65			0.0298 ± 0.0017	
FG_7d_		9.64 ± 0.51	0.24		12.62 ± 0.98	0.006		0.0386 ± 0.0032	0.01
CG_7d_		9.26 ± 0.86			13.92 ± 0.91			0.0425 ± 0.0031	

SD: standard deviation; p: level of statistical significance by Student’s t test.

### Liver regeneration rate

**Figure 2:**
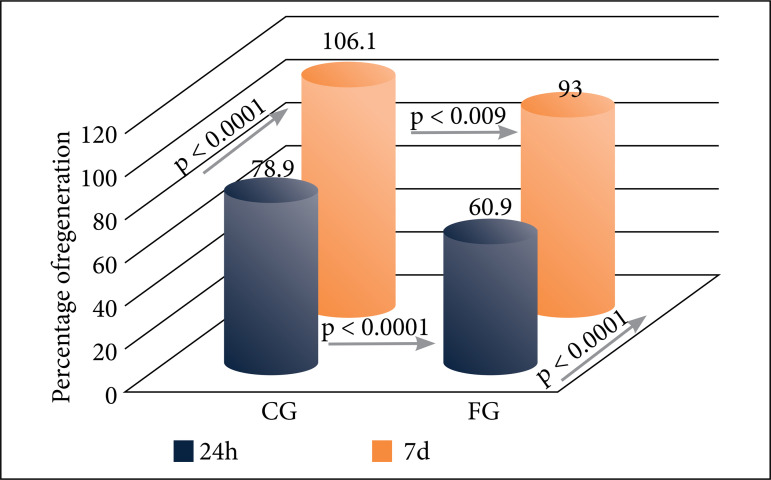
Liver regeneration percentages by Kwon formula. Statistical comparisons between the control (CG) and 5-fluorouracil (FG) groups at 24 h and 7 days. Blue: 24-h subgroups; Red: 7-day subgroups. p: Student’s t test.

Analysing hepatocytes mass changes throughout the experiment, it was possible to demonstrate significant differences between groups (fluorouracil effect) and inside groups (time effect). The assessment of liver regeneration using the Kwon formula showed a significant lower percentage of regeneration in the FG compared to that in the CG, for both 24-h subgroup (61% vs. 79%; p < 0.0001) and 7-day subgroup (93% vs. 106%; p = 0.009). There was also significant improvement in hepatic mass during time, with bigger hepatic remnants at 7 days compared to those at 24 h in both FG and CG subgroups (Fig. 2).

### Immunohistochemical analysis

**Figure 3:**
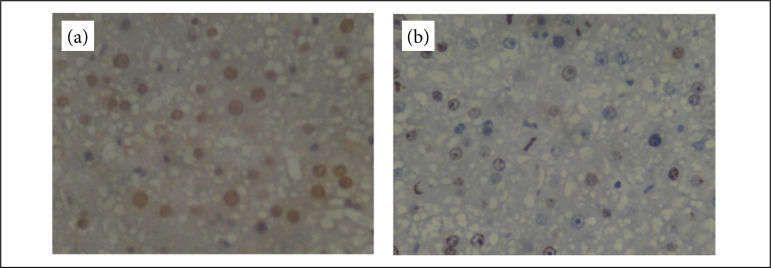
Microphotographs of immunohistochemical staining (brownish colour) for PCNA **(a)** and Ki-67 **(b)** in liver tissues of CG_7d_. Original magnification 400×.

The interference of 5-fluororacil on liver regeneration was assessed by immunohistochemistry for PCNA and Ki-67 antigen. Figure 3 shows representative PCNA and Ki-67 immunostains photomicrographs at 400× magnification of control subgroup 7 days. Following 70% hepatectomy, maximal PCNA expression occurred at 7 days, while maximal Ki-67 expression occurred at 24 h.

### Assessment of liver regeneration by PCNA

**Figure 4:**
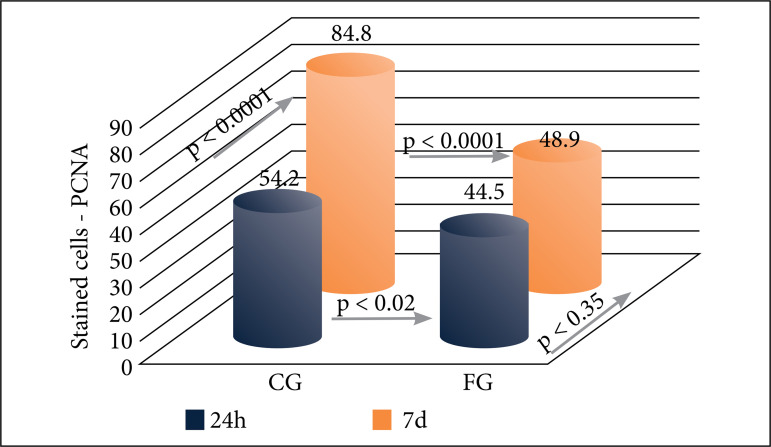
Liver regeneration by PCNA stained hepatocytes. Statistical comparisons between the control (CG) and 5-fluorouracil (FG) groups at 24 h and 7 days. Blue: 24-h subgroups; Red: 7-day subgroups. Mann–Whitney and Student’s t tests **(p)**.

The PCNA cell proliferation marker showed a significant difference in FG versus CG groups for both subgroups: 24 h (44.5 vs. 54.2; p = 0.02) and 7 days (48.9 vs. 84.8; p < 0.0001), with a lower percentage of stained cells in the FG subgroups during all the experiment. Comparing intragroups differences during the study, it was observed a continuous significant increment of stained cells in CG subgroups (24h vs. 7d; 54.2 vs. 84.8 respectively; p < 0.0001). The same comparison between FG subgroups did not show significant difference (24h vs. 7d; 44.5 vs. 48.9 respectively; p = 0.35) (Fig. 4).

### Assessment of liver regeneration by Ki-67

**Figure 5:**
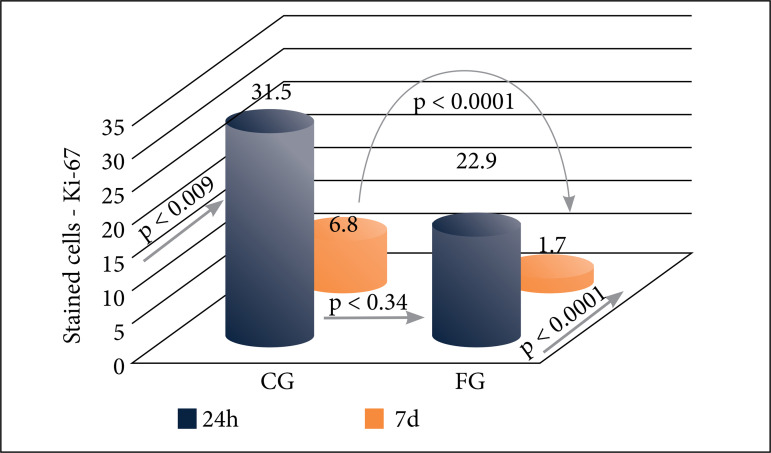
Liver regeneration by Ki-67-stained hepatocytes. Statistical comparisons between the control (CG) and 5-fluorouracil (FG) groups at 24 h and 7 days. Blue: 24-h subgroups; Red: 7-day subgroups. Mann–Whitney and Student’s t tests **(p)**.

The data concerning hepatocyte proliferation assessed by Ki-67 antigen, showed significant decreasing values occurring in both groups, from 24-h to 7 days analysis. At the higher expected moment of hepatocyte proliferation (24/36 h), equivalent values of Ki-67 antigen staining between subgroups (FG vs. CG; 22.9 vs. 31.5 respectively; p = 0.34) with no significant difference were found. When measured at final time (7 days), Ki-67 antigen staining showed marked decreasing values for both groups (FG and CG). However, at that time fluorouracil subgroup showed significant impairment in hepatocyte proliferation as compared to control subgroup (1.7 vs. 6.8; p < 0.0001) (Fig. 5).

## Discussion

In recent years, NAC treatment strategies combining new chemotherapy drugs and biological therapies have led to an increasing number of patients who can undergo liver resection due to CRC metastasis^2^^–^ 4. All these modern approaches were designed for increase control of toxic effects of chemotherapy drugs on liver parenchyma.

Although several studies^5^^,^ 8 ^,^ 9 have evaluated the toxic potential of chemotherapeutic agents on the liver parenchyma, the preoperative administration of chemotherapy is a relatively recent strategy and the effects of this approach on the regenerative potential of the liver remain unknown.

Therefore, there is a demanding investigative research for increased knowledge regarding the effect of these schemes on hepatic regenerative potential^12^, a subject of crucial importance for planning liver resections, particularly major resections and rehepatectomies^13^, where the possibility of postoperative liver failure must be considered.

Preoperative chemotherapy in the context of the clinical practice of treating CRC is administered before surgical treatment. In this study model, chemotherapy was administered in the prehepatectomy period to assess the effect of the drug on liver regeneration after hepatectomy. This methodology mimicked the use of chemotherapy as neoadjuvant therapy in humans.

However, results and conclusions available from clinical studies are based on retrospective analysis of series of patients who were treated with different doses of different drugs association during different periods of time, with chemotherapy being administered previously^14^^,^ 15 or even after the hepatectomy^13^. Moreover, most conclusions about the regenerated liver are particularly based on remnant liver volume measured by imaging exams^13^^–^ 15, a poor functional analysis.

The present study used a well-known and widely used experimental model of liver regeneration in rats, proposed by Higgins and Anderson^10^, and has the advantages of being easily reproducible, having a low cost, and not requiring complex structure and sophistication for its use.

The use of living models for the study of biological phenomena is of essential importance for understanding the complexity and interactions between the various elements involved in these phenomena as the regenerative processes of the liver result from a network of humoral, cellular, and molecular factors that generate initiators of cell division. For example, there is no DNA replication in cultured hepatocytes unless growth factors are added to the medium. Even so, the capacity for replication of hepatocytes in culture maintained under conventional conditions is very limited, differently from what occurs *in vivo* 11 ^,^ 16 ^,^ 17. Furthermore, as knowledge about liver regeneration and signal transduction grows, it is possible that some data has become therapeutic or modulating measures for posthepatectomy impaired liver regeneration. The recent data from the role of platelets on cell proliferation seems to be an example. Portal infusion of platelet-rich plasma into rats after 70% partial hepatectomy increased the liver/body weight ratio and the Ki-67 labelling index 24 h after liver resection^18^. Patients with preoperative platelet count less than 150,000/μL had higher incidence of posthepatectomy liver failure and mortality^19^. Therefore, evidence of the positive impact of platelets on liver regeneration from animal experiments and clinical studies anticipates the benefit of using platelets as prophylactic or therapeutic strategies for liver resection scenario^20^.

To assess the effect of preoperative chemotherapy on liver regeneration in rats, a single chemotherapeutic drug was used. In the oncological practice of treating CRC, chemotherapy drugs are generally used in combination, most commonly 5-fluorouracil with irinotecan or oxaliplatin. Specifically in NAC for hepatic CRC metastases, therapies with monoclonal antibodies, such as bevacizumab and cetuximab, in addition to others undergoing protocol evaluation, were administered with conventional chemotherapy to improve treatment response. Despite being one of the first chemotherapy drugs developed, 5-fluorouracil is still used in all first-line regimens in the treatment of CRC and in all NAC regimens for CRC metastases. For this reason, it was chosen in the present study to analyse separately its effect on the interference potential of post-hepatectomy regenerative phenomena.

The dose of 5-fluorouracil used, 20 mg/kg, is widely cited in studies of venous infusion in rats^21^^,^ 22 and represents a bioequivalent dose in humans. Although chemotherapy in humans is performed through various applications at varying intervals depending on the scheme, the use of multiple doses in rats is difficult and is associated with high mortality^22^^,^ 23. Although the use of a single dose of chemotherapy may be considered insufficient to affect liver regeneration after hepatectomy, in the present study it was sufficient to significantly alter some parameters used to assess regeneration at two different time points after major hepatectomy.

According to the day of sacrifice, liver samples were collected 24 h or 7 days after surgery. This strategy is essential for the assessment of liver regeneration in rats since the regenerative phenomena begin minutes after hepatectomy and most of the fundamental physiological mechanisms are established within 24 h.

Analysis of the weights of the animals showed significant weight loss in the 24-h subgroups (CG and FG) in the interval between the day of administration of the solutions and the day of death. The 7-day subgroups (CG and FG) did not show weight loss in the same comparison. These results are explained by the fact that 7 days after hepatectomy is sufficient to recover from surgical stress and permit weight gain in these animals. However, no significant difference in body weight was found between control and fluorouracil subgroups throughout the experiment, demonstrating no interference of chemotherapy on lost body weight.

The estimated liver weight at the time of hepatectomy (24 h after chemotherapy) showed a significant increase in liver weight of the animals receiving 5-fluorouracil comparing to CG (Table 2). Curiously, animals’ body weight showed the same trend at same time (Table 1), which could indicate a systemic inflammatory condition after chemotherapy. The regenerated liver, weighted 24 h or 7 days after hepatectomy, always showed significant lower values for animals receiving chemotherapy when compared to CG (Table 2), suggesting interference of 5-fluorouracil on the regenerative potential since liver weight is considered a parameter of the hepatic regenerative capacity. Identical results were obtained from the analysis of the ratio of regenerated liver weight/body weight supporting a delay effect of 5-fluorouracil on liver regeneration.

When assessing liver regeneration using the Kwon formula^11^, the statistical analysis revealed a significant delay in the hepatic regenerative potential for FG subgroups compared to the CG subgroups. At 24 h after hepatectomy, the average percentage of regeneration in the CG group was 78.9% compared to 60.9% in the FG group; after 7 days, the percentage of regeneration in the CG was 106.1% compared to 93% in the FG (Fig. 2). These differences suggest the interference of 5-fluorouracil on liver regeneration.

This fast and incredible regenerative capacity in rats has been well studied. As reported by Fausto *et al*.^24^, mitosis begins quickly after hepatectomy, peaking at 32–34 h after surgery. Additional cycles of DNA synthesis can be detected days after partial hepatectomy^24^^,^ 25 which explained the percentage increase in liver regeneration in the 7-day groups and the significant difference in the comparisons between the 24-h and 7-day groups.

The cell proliferation markers PCNA and Ki-67 were also used to assess liver regeneration. The PCNA analysis revealed a significant difference between the FG and CG at 24 h and 7 days, with a lower percentage of stained cells in the 5-fluorouracil groups, suggesting the interference of 5-fluorouracil on the hepatic regenerative potential in both periods. PCNA was not expected to show high rates over 7-day periods compared to 24-h periods. The possible explanations for this effect may be related to the fact that the rats used in the study were young adults; thus, with a high capacity for regeneration. In addition, only two time points were used to assess the effects of 5-fluorouracil on liver regeneration, which may have limited the evaluation.

The analysis of Ki-67 staining confirmed the higher number of stained cells in the 24-h subgroups compared to those in the 7-day subgroups, indicating the greater regenerative potential, that is, a greater number of cells outside the G0 phase in the first 24 h after hepatectomy. No significant difference was observed between the CG and FG subgroups at 24 h but a significant difference was observed between the CG and FG subgroups at 7 days, suggesting a negative effect of 5-fluorouracil in the late phase of liver regeneration.

A small number of experimental studies have assessed the effect of 5-fluorouracil in the neoadjuvant scenario, probably due to the relatively recent neoadjuvant administration scheme in humans. However, studies have proven the suppressive effect of liver regeneration when 5-fluorouracil is administered in the post-hepatectomy period^23^^,^ 25 ^,^ 26.

Despite these findings, as well as those of Manekeller *et al*.^23^, regarding the negative effects of preoperative administration of chemotherapeutic agents on the potential for liver regeneration, another study showed different results. Rickenbacher *et al*.^27^ administered several cycles of combinations of 5-fluorouracil with irinotecan and 5-fluorouracil with oxaliplatin administered intraperitoneally in rats and did not observe any interference of neoadjuvant schemes in the hepatic regenerative potential by immunohistochemical analysis of PCNA and Ki -67. Comparison of these results to the present study is difficult since both the route of administration and the chemotherapy regimens differed between the two studies.

When analysing the NAC context in clinical practice, one should consider the fact that 5-fluorouracil is always administered in association with other drugs and in several cycles. Such treatments cause damage to the liver parenchyma, as also mentioned in the present study. However, it is not clear whether this damage diminishes the liver’s ability to regenerate after extensive resection. It is difficult to reproduce the current treatments at an experimental level since the intravenous administration of chemotherapy for several cycles in rats becomes complex due to technical difficulties.

Therefore, one must consider the limitations of this experimental model, in the sense of reproducing in rats the histological condition of the human liver parenchyma after several cycles of chemotherapy.

Additional studies are needed to explore chemotherapy administered intravenously in several cycles, since the toxicity of chemotherapy on the liver parenchyma may be related to late histological damage after several cycles of chemotherapy and dependent on the different schemes used. For this purpose, systematic review protocols may be of help, particularly if they can address the effect of different preoperative chemotherapy schemes on liver parenchyma submitted to portal vein ligation and/or major resection during colorectal liver metastasis treatment^12^.

Thus, the successful use in this study of the 5-fluorouracil chemotherapy alone expands the landscape of experimental possibilities for follow-up in the investigation of the effects of preoperative chemotherapy on liver regeneration with the use of other drugs and associations, thus contributing to the knowledge of the effects of these treatments on liver regeneration.

## Conclusion

Preoperative chemotherapy with single dose of intravenous 5-fluorouracil is capable to negatively affect the early and late mechanisms of liver regeneration after major hepatectomy in rats. Additional experimental data with different drugs and schemes of chemotherapy would be welcome. The present and future data must be taken into account in the surgical planning of therapeutic strategies for human liver tumours/metastases in order to obtain the best results and reduce morbidity and mortality, particularly in the NAC scenario.
